# Analysis of multicenter clinical trials with very low event rates

**DOI:** 10.1186/s13063-020-04801-5

**Published:** 2020-11-09

**Authors:** Jiyu Kim, Andrea B. Troxel, Scott D. Halpern, Kevin G. Volpp, Brennan C. Kahan, Tim P. Morris, Michael O. Harhay

**Affiliations:** 1 Division of Biostatistics, Department of Population Health, New York University Grossman School of Medicine, New York, NY USA; 2 Palliative and Advanced Illness Research (PAIR) Center, Department of Medicine, Perelman School of Medicine, University of Pennsylvania, 304 Blockley Hall, 423 Guardian Drive, Philadelphia, PA 19104-6021 USA; 3 Division of Pulmonary, Allergy, and Critical Care, Department of Medicine, Perelman School of Medicine, University of Pennsylvania, Philadelphia, PA USA; 4 Department of Biostatistics, Epidemiology, and Informatics, Perelman School of Medicine, University of Pennsylvania, Philadelphia, PA USA; 5 Department of Medical Ethics and Health Policy, Perelman School of Medicine, University of Pennsylvania, Philadelphia, PA USA; 6 Center for Health Incentives and Behavioral Economics, Perelman School of Medicine, University of Pennsylvania, Philadelphia, PA USA; 7 Philadelphia VA Medical Center, Philadelphia, PA USA; 8 Department of Health Care Management, Wharton School, University of Pennsylvania, Philadelphia, PA USA; 9 MRC Clinical Trials Unit at UCL, London, UK; 10 Department of Medical Statistics, London School of Hygiene and Tropical Medicine, London, UK

**Keywords:** Multicenter trial, Binary outcomes, Low event rate, Randomized clinical trial, Random effects, GEE, Small sample adjustment, Mantel–Haenszel, Stratified randomization

## Abstract

**Introduction:**

In a five-arm randomized clinical trial (RCT) with stratified randomization across 54 sites, we encountered low primary outcome event proportions, resulting in multiple sites with zero events either overall or in one or more study arms. In this paper, we systematically evaluated different statistical methods of accounting for center in settings with low outcome event proportions.

**Methods:**

We conducted a simulation study and a reanalysis of a completed RCT to compare five popular methods of estimating an odds ratio for multicenter trials with stratified randomization by center: (i) no center adjustment, (ii) random intercept model, (iii) Mantel–Haenszel model, (iv) generalized estimating equation (GEE) with an exchangeable correlation structure, and (v) GEE with small sample correction (GEE-small sample correction). We varied the number of total participants (200, 500, 1000, 5000), number of centers (5, 50, 100), control group outcome percentage (2%, 5%, 10%), true odds ratio (1, > 1), intra-class correlation coefficient (ICC) (0.025, 0.075), and distribution of participants across the centers (balanced, skewed).

**Results:**

Mantel–Haenszel methods generally performed poorly in terms of power and bias and led to the exclusion of participants from the analysis because some centers had no events. Failure to account for center in the analysis generally led to lower power and type I error rates than other methods, particularly with ICC = 0.075. GEE had an inflated type I error rate except in some settings with a large number of centers. GEE-small sample correction maintained the type I error rate at the nominal level but suffered from reduced power and convergence issues in some settings when the number of centers was small. Random intercept models generally performed well in most scenarios, except with a low event rate (i.e., 2% scenario) and small total sample size (*n* ≤ 500), when all methods had issues.

**Discussion:**

Random intercept models generally performed best across most scenarios. GEE-small sample correction performed well when the number of centers was large. We do not recommend the use of Mantel–Haenszel, GEE, or models that do not account for center. When the expected event rate is low, we suggest that the statistical analysis plan specify an alternative method in the case of non-convergence of the primary method.

**Supplementary information:**

**Supplementary information** accompanies this paper at 10.1186/s13063-020-04801-5.

## Introduction

Multicenter randomized clinical trials (RCTs) that enroll participants from multiple settings (e.g., countries, hospitals, clinics, or villages; hereafter “centers”) are increasingly common in contemporary health and social sciences research. This is primarily because they hasten and increase total recruitment while promoting the generalizability of trial results. However, enrollment at multiple locations can introduce variation in participant composition and outcomes among centers as well as disproportional enrollment across centers. As such, multicenter trials sometimes use center as a stratification factor in the randomization procedure to ensure that an equal number of participants is allocated to each study group within each center. When randomization is stratified by a study center (or any other factor), it is necessary to account for this in the trial analysis [1, 2]. If the stratification factors are not accounted for, the resulting standard errors (SEs) of the treatment effect estimate can be biased upwards, leading to *p* values that are too large and confidence intervals (CIs) that are too wide, effectively reducing statistical power.

**Fig. 1:**
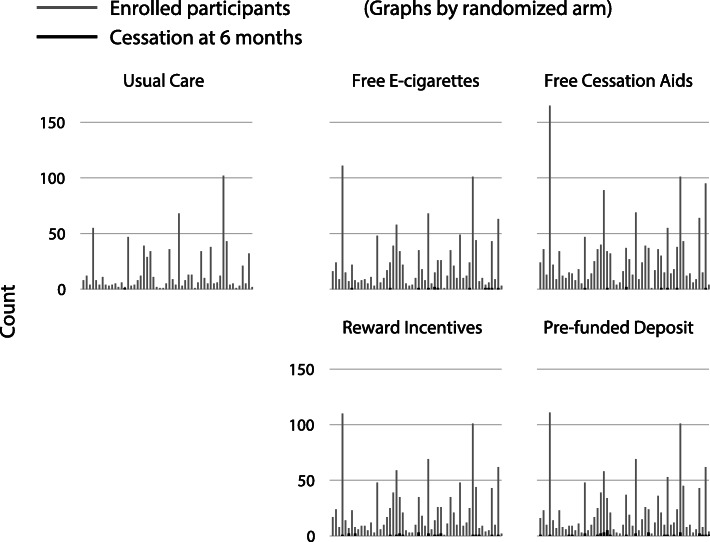
Distribution of participants who were randomized (light gray) and who had sustained cessation (black) from smoking for 6 months, across 54 companies in the motivating five-arm randomized trial of workplace smoking cessation programs [3]. A total of 6006 participants were randomized, and 80 achieved sustained cessation, resulting in several companies with no participants quitting either overall or in more than one study arm

The focus of this paper is the analysis of multicenter trials with stratified randomization by center and a binary outcome with extremely low outcome event rates. When there are very low event rates, it is likely that there will be some centers where no participants experience an event, or only participants in one study group experience an event, which poses a challenge for the statistical analysis. This question was motivated by a recent RCT in which we enrolled 6006 smokers from 54 companies (i.e., centers) to test four workplace-based smoking cessation interventions against usual care [3]. The participating companies were of different underlying sizes and contributed a median of 59 participants (range, 6 to 552; interquartile range, 38 to 130; Fig. 1). Due to expected correlations among individuals within each company (and notable differences between companies), we stratified the randomization by company. Therefore, adjustment for company in the analysis was deemed necessary and was part of the initial statistical analysis plan. Our study design predicted 6-month smoking cessation proportions of 2.5% in the usual care arm and 7.5% in the intervention arms. At the end of the trial, the number of individuals who ceased smoking for 6 months was much lower than expected in all arms, with only 80 total individuals having quit smoking (1.3%). The cessation percentages across the five arms were 0.1%, 0.5%, 1.0%, 2.0%, and 2.9%. As a result, many companies had zero individuals who quit either overall or in one or more study arms (Fig. 1).

This example highlights a statistical tension. Some common methods of adjustment for center, such as fixed-effects models or Mantel–Haenszel methods, will exclude participants from the analysis if they belong to a center in which no participants in a certain treatment arm experienced an event or all participants in that center were allocated to the same treatment arm. This exclusion of participants can lead to a loss of statistical power and precision and directly contradicts the intention-to-treat principle. Thus, the goal of this paper is to provide practical guidance on the optimal statistical method for odds ratio estimation with center adjustment when randomization has been stratified by center and the outcome event proportions may be extremely low. To accomplish this aim, we sought to identify the statistical solution that would meet certain pre-established criteria. Specifically, we sought to identify a method that (i) does not drop any participants from the analysis unnecessarily, adhering to the intention-to-treat principle; (ii) has a high probability of reaching convergence; (iii) gives an unbiased estimation of the SE so as to achieve appropriate CI coverage and type I error rate; and (iv) maximizes statistical power.

Previous work has discussed options for the analysis of multicenter trials with low event rates [4] and empirically evaluated methods of analysis with binary outcomes with moderate event rates using simulation [5–7]. However, to our knowledge, there have been no studies that have empirically evaluated common methods of estimating an odds ratio in multicenter trials with low event rates. We therefore conducted a simulation study to compare these methods across a range of possible trial scenarios. We begin with a description of methods that are commonly used in practice and then present a statistical simulation study to evaluate these methods. We then present a reanalysis of the motivating RCT using the selected methods. Finally, we summarize and provide practical recommendations for researchers.

## Methods

In a multicenter trial, the variance of the treatment effect estimate is influenced by the intra-class correlation coefficient (ICC), which indicates the similarity of outcomes of participants within a cluster relative to those in other clusters. In other words, the ICC measures the percentage of the total variability explained by different centers and can be written as ${\sigma}_c^2/\left({\sigma}_c^2+{\sigma}_{\varepsilon}^2\right)$, where ${\sigma}_c^2$ is between-center variance and ${\sigma}_{\varepsilon}^2$ is the variance of the random error. It is well known that ignoring site-specific correlation in the analysis after randomization has been stratified by center can reduce power proportional to the ICC value [1]. Kahan [5] suggested adjusting for center effects to ensure correct *p* values and to avoid a loss of power when randomization is stratified by center. In addition, even if center is not a stratification factor, we might adjust for center effects to increase power when the ICC is expected to be large.

To adjust for center effects, we can use either a marginal or conditional model. These models target different treatment estimands (i.e., marginal vs. conditional estimand), and for the odds ratio, the true value of these estimands is different in general [8, 9]. If the treatment effect is null or the ICC is 0, the true value of the estimands is equal. As the ICC increases, so too will the difference between the estimands’ values. These estimands have different interpretations; marginal estimands compare the change in odds for participants across centers, whereas conditional estimands compare the change in the odds for participants within the same center [8, 9].

In this paper, we examined five methods of analysis: one approach that ignores center in the analysis, as well as four commonly used statistical models that adjust for center. The five models are (i) logistic regression model with no adjustment for center (unadjusted), (ii) mixed-effects logistic regression model with a random intercept for center (RE), (iii) the Mantel–Haenszel method, (iv) generalized estimating equation (GEE) with an exchangeable correlation structure across centers and robust SEs, and (v) GEE with small sample correction (GEE-small sample correction). Of note, we did not examine fixed-effect models (where each center is included as a covariate in the model using indicator variables), as these methods have previously been shown to perform poorly for binary outcomes with low event rates [5], and they exclude participants in centers with no events. In particular, the use of fixed effects is inappropriate in the context of our motivating example and simulation settings, as the number of parameters in the model would approach or exceed the number of events. The problem of participant exclusion from centers with no events is also a limitation of the Mantel–Haenszel approach, but because this method is commonly taught as a way to estimate overall and stratified odds ratios, we felt it would be useful to examine its statistical performance. Finally, we did not consider the issue of treatment-by-center interaction and assumed that the odds ratio for treatment was the same across all centers.

### Center-unadjusted logistic regression model

A center-unadjusted logistic model targets a marginal estimand and can be written as:

$$ {y}_{ij}\sim \mathrm{Bernoulli}\left({p}_{ij}\right) $$

$$ \mathrm{logit}\left({\pi}_{ij}\right)=\alpha +{\beta}_{\mathrm{trt}}{X}_{ij} $$

where *y*_*ij*_ is the binary outcome for the *i*th participant in the *j*th center with *p*_*ij*_ = *P*(*Y*_*ij*_ = 1), *π*_*ij*_ = *p*_*ij*_/(1 − *p*_*ij*_), *X*_*ij*_ is a treatment indicator, and the coefficient *β*_trt_ is the log odds ratio for treatment. Additional continuous or categorical predictor variables can be included in the model. In our case of not accounting for center effects, the model includes only the treatment indicator, *X*_*ij*_.

### Center adjusted models

#### Conditional models

##### Mixed-effects logistic regression model with a random intercept for center

The random intercept model can be written as:

$$ \mathrm{logit}\left({\pi}_{ij}\right)=\alpha +{\beta}_{\mathrm{trt}}{X}_{ij}+{u}_j $$

where the notation is defined as above and *u*_*j*_ is a random intercept for the *j*th center, generally assumed to be distributed *u*_*j*_~N (0, *σ*^2^). The random intercept model includes both fixed (*β*_trt_) and random (*u*_*j*_) components. The parameter *σ*^2^ provides a summary of the variation among centers. A random intercept model is able to include participants from all centers in the analysis, even when all participants are randomized to the same group or all participants in a treatment group within a center experience the same outcome. Previous studies have shown that random-effects models provide unbiased estimates of the treatment effect, preserve the type I error rate at its nominal level, and have good power [5, 6]. However, they have not been evaluated in settings with extremely low event rates.

##### Mantel–Haenszel

The Mantel–Haenszel approach calculates an odds ratio within each center and then generates a combined common odds ratio by weighting the center-specific odds ratios according to the number of participants in each center. For a 2 × 2 × *J* table, where *j* = 1, …, *J* indexes centers, the estimate of the common odds ratio is:

$$ {\mathrm{OR}}_{\mathrm{MH}}=\frac{\sum_j\frac{a_j{d}_j}{n_j}}{\sum_j\frac{b_j{c}_j}{n_j}} $$

where *a*_*j*_ and *b*_*j*_ indicate the number of participants with and without an event in the treatment group, respectively; *c*_*j*_ and *d*_*j*_ indicate the number of participants with and without an event in the control group, respectively; and *n*_*j*_ is the total number of participants in the *j*th center. Mantel–Haenszel models exclude participants from centers where all participants are randomized to the same group or all participants in a treatment group within a center experience the same outcome. As such, Mantel–Haenszel methods can lead to lower power and bias when many participants are excluded from the analysis [4, 5].

#### Marginal models

##### Generalized estimating equation

A logistic regression model using GEE is written as:

$$ \mathrm{logit}\left({\pi}_{ij}\right)=\alpha +{\beta}_{\mathrm{trt}}{X}_{ij} $$

To obtain the GEE estimates, a working correlation structure must be specified (e.g., independent, exchangeable). Following Liang and Zeger [10], the estimates $\hat{\beta}$ can be obtained by solving the estimating equation ${\sum}_{i=1}^K{D}_i^{\prime }{V}_i^{-1}\left({Y}_i-{\mu}_i\right)=0$, where ${D}_i=\frac{\partial {\mu}_i}{\partial {\beta}^{\prime }}$, and the variance of $\hat{\beta}$ is estimated by:

$$ V=\varOmega \left(\sum \limits_{i=1}^K{D}_i^{\prime }{V}_i^{-1}\hat{r_i}{\hat{r_i}}^{\prime }{V}_i^{-1}{D}_i\right)\varOmega, $$

where $\varOmega ={\left({\sum}_{i=1}^K{D}_i^{\prime }{V}_i^{-1}{D}_i\right)}^{-1},$ and $\hat{r_i}{\hat{r_i}}^{\prime }$=$\hat{\mathrm{Cov}\left({Y}_i\right)}$, with $\hat{r_i}={Y}_i-\hat{\mu_i}$. This variance–covariance estimator is called the “sandwich” or “robust” estimator and is commonly used to calculate SEs for GEEs. It is a consistent estimator of the true underlying variance–covariance matrix even if the correlation structure is incorrectly specified. In our simulation study, we used robust SE estimators and chose the exchangeable correlation as a working correlation structure, which assumes that all outcomes within a center are equally correlated, corr(*Y*_*ij*_, *Y*_*ik*_) = *ρ*, *j* ≠ *k*.

##### GEE with small sample correction

The robust variance estimator is often employed in GEE models because it produces unbiased SE estimates for regression coefficients even when the covariance structure is mis-specified. However, the robust covariance matrix estimator relies on asymptotic theory, which assumes a large number of centers. Thus, the robust SEs typically reported for GEEs are downward-biased when the number of centers is small, leading to an inflated type I error rate [11, 12]. This fact restricts the application of GEEs in settings with a small number of centers. Therefore, several small sample corrections for GEEs have been proposed to improve its performance with a small or medium number of centers [13, 14]. These methods adjust the estimated variance without affecting the estimated treatment effect. The Fay and Graubard correction has been shown to perform well with a small number of clusters [13], and so in our simulation study, we applied their correction [14] to evaluate its performance in terms of the type I error rate and power. Fay and Graubard suggested the corrected sandwich estimator, defined as:

$$ {V}_{\mathrm{FG}}=\varOmega \left(\sum \limits_{i=1}^K{c}_i{D}_i^{\prime }{V}_i^{-1}\hat{r_i}{\hat{r_i}}^{\prime }{V}_i^{-1}{D}_i{c}_i\right)\varOmega, $$

where ${c}_i=\Big\{1-\min {\left(b,{\left\{{D}_i^{\prime }{V}_i^{-1}{D}_i\Omega \right\}}_{jj}\right)}^{-1/2}$ and *b* is a constant bound for bias correction defined by the user; it should be less than 1 to prevent extreme adjustments when element *j*,*j* of $\left\{{D}_i^{\prime }{V}_i^{-1}{D}_i\varOmega \right\}$ is very close to 1 [14]. Fay and Graubard arbitrarily used *b* = 0.75 in simulations, achieving almost exactly the same results when run without the bound *b*. We also set *b* = 0.75 (the default in R) in this simulation study.

### Simulation study

We considered a multicenter clinical trial with *J* centers where participants were randomized to a treatment or control group using a 1:1 allocation ratio. To cover a variety of study designs, we varied several parameters, including the number of centers, total number of participants, ICC, control arm event proportion (probability), and treatment effect, as summarized in Table 1.

**Table 1:** Trial settings and parameters examined in the statistical simulation study

*Trial design characteristics*	*Settings*
Total sample size	200, 500, 1000, and 5000
Number of centers	5, 50, and 100
ICC	0.025 and 0.075
Control group event probability	0.02, 0.05, and 0.10
Distribution of participants across centers	Balanced and skewed
True odds ratio	1 and > 1
Randomization ratio	1:1
Permuted block size	4

#### Methods of analysis

All simulations were performed using R 3.5.1 [15]. We used the “glm” function to fit the unadjusted model (which ignored center) and the “glmer” function in the “lme4” [16] package to fit the random intercept (RE) model, with the number of points per axis for evaluating the adaptive Gauss–Hermite approximation set to 9. The “mantelhaen.test” function was used to fit the Mantel–Haenszel method. GEE models were implemented using the “geeglm” function in the package “geepack” [17], and GEE with small sample correction was estimated using the “saws” [14] package. Sample R code is provided in the online supplement.

#### Data-generating mechanism

For each setting, we simulated 5000 hypothetical trial datasets, expecting small error due to the large number of repetitions. The choice of 5000 repetitions was arbitrary but justified by sufficiently low Monte Carlo errors. The datasets for each combination of parameters were generated from the following model:

$$ {Y}_{ij}^{\ast }=\alpha +{\beta}_{\mathrm{trt}}{X}_{ij}+{u}_j+{\varepsilon}_{ij} $$

where ${Y}_{ij}^{\ast }$ is a latent variable generated by given parameters. The treatment indicator *X*_*ij*_ was generated using permuted blocks stratified by center with a block size of 4. A random center effect *u*_*j*_ was generated from a normal distribution with a mean of 0 and standard deviation *σ*, which was set by the desired ICC (on the logistic scale), and *ε*_*ij*_ was a random error from the standard logistic distribution. The binary outcome *Y*_*ij*_ was generated as 1 if the latent variable ${Y}_{ij}^{\ast }$ was greater than 0, and as 0 otherwise.

The number of centers in each setting was either 5, 50, or 100, and the total number of participants randomized was 200, 500, 1000, or 5000. The ICC was set to either 0.025 or 0.075 (on the logit scale), and we used two different scenarios for the distribution of participants across the centers (balanced or skewed). First, we set each center to have an equal number of participants (balanced). Second, we set each center to have a different number of participants (skewed). Every time we generated a sample dataset, the number of participants in each center was generated using a multinomial distribution while fixing the total number of participants. An example distribution of participants is provided in Table 2. The actual number of centers sometimes differed slightly from the target number of centers (i.e., 5, 50, or 100) when the participant distribution was skewed, especially when the number of participants was relatively small (i.e., 200 or 500) compared to the number of centers (i.e., 100). We reported the actual number of centers in the results. The overall event probability was 0.02, 0.05, or 0.10 in the control group. We also set the true odds ratio for treatment to 1 to evaluate the type I error rate. To evaluate statistical power, the true conditional odds ratio was chosen in order to provide 80% power based on the given total number of participants and the event rate in the control group. The true odds ratio was set based on an increase in events (denoting a beneficial outcome), meaning that the true odds ratio was set to be > 1 in these scenarios. Table S1 in the supplemental material provides the true conditional odds ratio for each scenario. We calculated the true marginal odds ratio as ${\beta}_{\mathrm{marginal}}\approx \frac{\beta_{\mathrm{conditional}}}{0.014}$ for ICC = 0.025 and as ${\beta}_{\mathrm{marginal}}\approx \frac{\beta_{\mathrm{conditional}}}{0.045}$ for ICC = 0.075 [18]. In our simulation study, we compared estimates from conditional models (Mantel–Haenszel and random intercept [RE]) against the true conditional odds ratio, and estimates from marginal models (GEE, GEE-small sample correction, center-unadjusted model) against the true marginal odds ratio.

**Table 2:** Example of a skewed participant distribution with 5 centers and a total sample size of 500 participants

	Center 1	Center 2	Center 3	Center 4	Center 5
Number of participants	113	84	102	97	104

In some repetitions, we encountered model convergence issues, such that the simulation would not progress. As a solution, we set up a prescreening approach that excluded from the analysis any data-generating process that created a simulated trial with (i) no events or (ii) all events occurring in the same treatment group.

#### Estimand

The estimand of interest is the true odds ratio (the true conditional odds ratio for conditional models and the true marginal odds ratio for marginal models).

#### Performance measures

For each simulation scenario and model, we estimated the following measures (along with a Monte Carlo SE for each):

1. Type I error rate (when the true odds ratio is 1)
2. Power (when the true odds ratio is > 1)
3. Estimated odds ratio
4. Convergence rate
5. Coverage rate of 95% CIs

Type I error rate and power were calculated as the proportion of the simulation results with a statistically significant treatment effect with a two-sided significance level of 5%. The coverage of 95% CIs was estimated as the proportion of the simulation results in which the estimated 95% CI contained the true value of the odds ratio. The mean value of the estimated treatment effect (i.e., odds ratio) was calculated by exponentiating the mean of the estimates of *β*_trt_. The Monte Carlo SE for each performance measure was also provided as an estimate of simulation uncertainty [19]. In calculating performance, we included only the repetitions in which the model successfully converged. The model was classified as a convergence failure when (i) we received an error or warning message indicating the analysis did not converge or (ii) the absolute value of either the odds ratio for treatment or the SE was greater than 1000. We chose this approach for non-convergence as there is no clearly preferable alternative. For instance, counting non-converged replications in the numerator or denominator could be misleading as it conflates convergence issues with statistical issues such as bias or the type I error rate. In addition, we believed that the potential for non-convergence was an important metric for researchers when assessing model options for a trial.

### Reanalysis of the motivating trial

To examine the impact of the five methods assessed in the statistical simulation study in an actual multicenter trial with stratified randomization, we undertook a reanalysis of the motivating five-arm smoking cessation trial [3]. For this analysis, we used the same commands as used in the statistical simulation study. For each approach, we report the sample size, odds ratios, and 95% confidence intervals of the effect estimate using the e-cigarettes group as the reference group.

## Results

### Computational issues

We encountered issues related to convergence, particularly with the “gee” function in R. This was the most problematic in settings with a skewed participant distribution across centers and when both the number of centers and total participants were small. Because the “gee” function is used for the GEE-small sample correction method in R, there were several replications where we could not obtain estimates in certain settings because of convergence failure. We also examined these datasets using the “xtgeebcv” [20] package in Stata, where convergence also sometimes failed. As noted above, we encountered issues with model convergence with the Mantel–Haenszel approach in similar settings. Unadjusted logistic models, RE, and GEE (“geeglm” function) generally had high convergence rates.

### Simulation study

Results for type I error, power, estimated odds ratio, coverage of 95% CI, and convergence rate are shown in Figs. 2, 3, 4, 5, and 6, respectively. For ease, we summarize the key results of the simulation study here. We also provide a narrative summary of results by the total number of centers in the online supplement for researchers who are considering studies with a fixed number of sites. In addition, Supplemental Figures S1 to S15 in the online supplement summarize additional results from the simulation study.

**Fig. 2:**
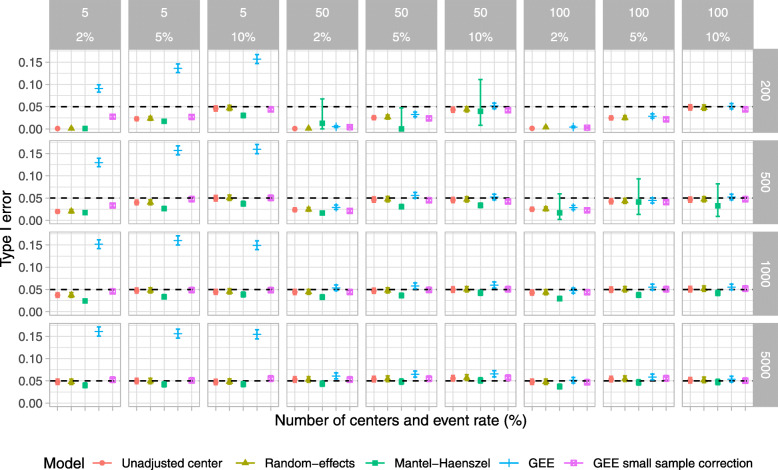
Type I error rates for scenarios when the number of centers was 5, 50, and 100 and the total number of participants was 200, 500, 1000, and 5000. The ICC was fixed at 0.025, and the participant distribution was skewed across the centers. *The value for the Mantel–Haenszel model was not available because of convergence issues when the number of centers was 100 and the number of total participants was 200

**Fig. 3:**
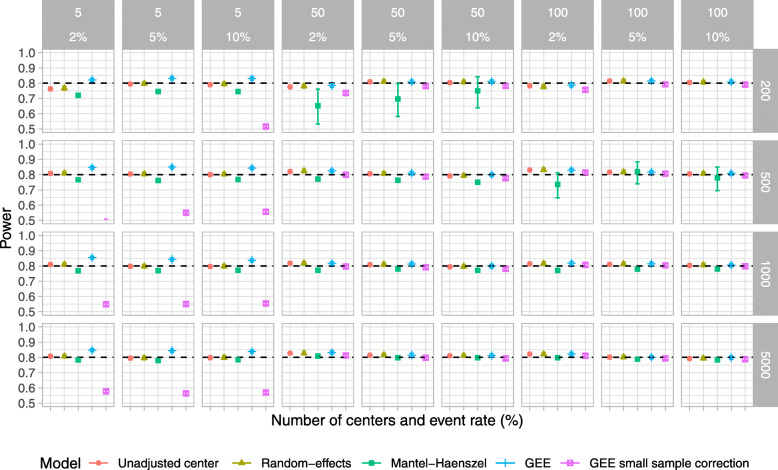
Power for scenarios when the number of centers was 5, 50, and 100 and the total number of participants was 200, 500, 1000, and 5000. The ICC was fixed at 0.025, and the participant distribution was skewed across the centers. *The value for the Mantel–Haenszel model was not available because of convergence issues when the number of centers was 100 and the number of total participants was 200

**Fig. 4:**
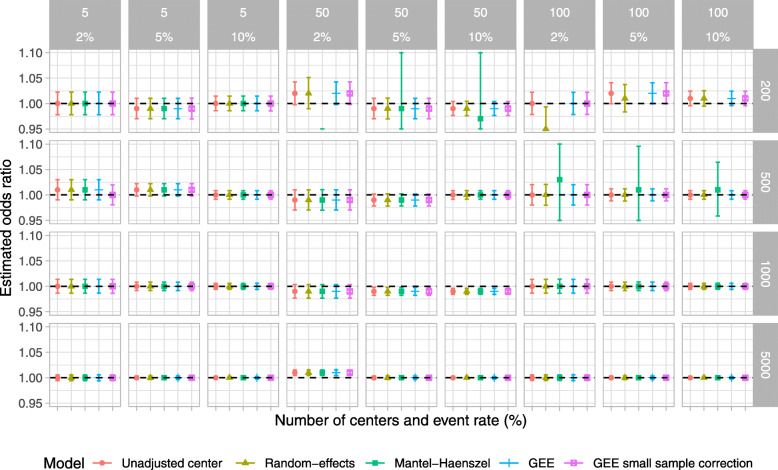
Estimated mean treatment odds ratio (OR) for scenarios when the true odds ratio was 1; the number of centers was 5, 50, and 100; and the total number of participants was 200, 500, 1000, and 5000. The ICC was fixed at 0.025, and the participant distribution was skewed across the centers. *The value for the Mantel–Haenszel model was not available because of convergence issues when the number of centers was 100 and the number of total participants was 200

**Fig. 5:**
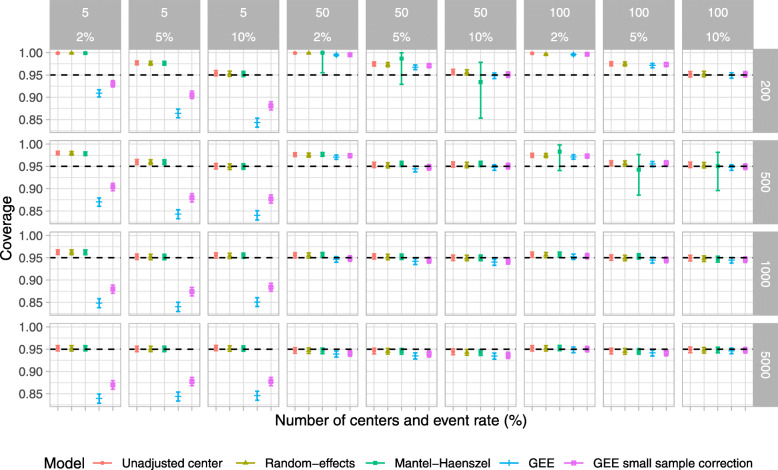
Coverage of 95% confidence intervals for scenarios when the true odds ratio was 1; the number of centers was 5, 50, and 100; and the total number of participants was 200, 500, 1000, and 5000. The ICC was fixed at 0.025, and the participant distribution was skewed across the centers. *The value for the Mantel–Haenszel model was not available because of convergence issues when the number of centers was 100 and the number of total participants was 200

**Fig. 6:**
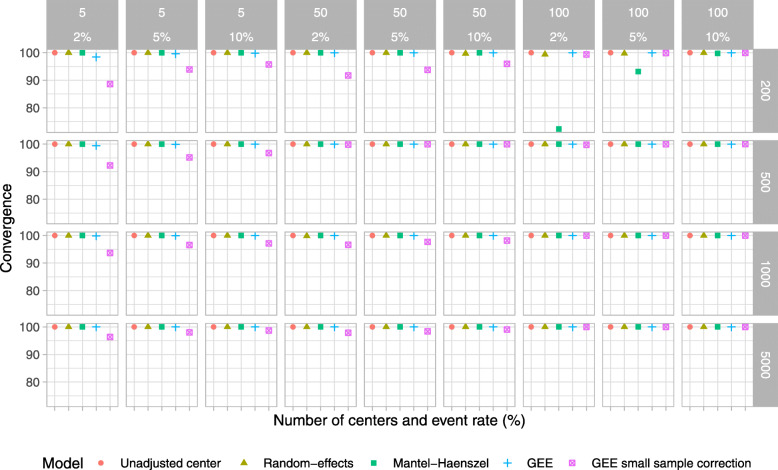
Convergence for scenarios when the true odds ratio was 1; the number of centers was 5, 50, and 100; and the total number of participants was 200, 500, 1000, and 5000. The ICC was fixed at 0.025, and the participant distribution was even across the centers

Overall, when the sample size was small (*n* = 200 and 500) and the event proportion was low (2%), all of the examined methods produced biased odds ratios, type I error rates that were below the nominal level, and low power. The model that did not account for center had a lower than nominal type I error rate and reduced power compared to RE, GEE, or GEE-small sample correction, particularly when the ICC was 0.075.

The RE model performed well in most other scenarios and performed as well or better than other methods in terms of the nominal type I error rate, power, and unbiased estimates in most scenarios (Tables 3 and 4). The one exception was when there were 100 centers and 200 total participants, when RE models gave slightly biased estimates when the true odds ratio was greater than 1.

**Table 3:** Type I error, mean estimated odds ratio (OR), convergence rate (%), and coverage of 95% confidence intervals when the number of centers was 5, the ICC was 0.025, the event rate was 10%, the participant distribution across centers was skewed, and the true OR was 1

Number of participants (***n***)	Measurement	Unadjusted	RE	MH	GEE	GEE-small sample correction
*n* = 200	Type I error	0.046	0.047	0.030	0.157	0.044
	MCSE (type I error)	0.003	0.003	0.002	0.005	0.003
	Mean	1	1	1	1	1
	MCSE (mean)	0.007	0.007	0.007	0.007	0.007
	Convergence	100	100	100	99.4	97.08
	Coverage	0.954	0.953	0.953	0.843	0.881
	MCSE (coverage)	0.003	0.003	0.003	0.005	0.005
*n* = 500	Type I error	0.049	0.050	0.037	0.160	0.050
	MCSE (type I error)	0.003	0.003	0.003	0.005	0.003
	Mean	1	1	1	1	1
	MCSE (mean)	0.004	0.004	0.004	0.004	0.004
	Convergence	100	100	100	99.9	99.08
	Coverage	0.951	0.950	0.95	0.840	0.877
	MCSE (coverage)	0.003	0.003	0.003	0.005	0.005
*n* = 1000	Type I error	0.044	0.045	0.039	0.149	0.048
	MCSE (type I error)	0.003	0.003	0.003	0.005	0.003
	Mean	1	1	1	1	1
	MCSE (mean)	0.003	0.003	0.003	0.003	0.003
	Convergence	100	100	100	99.98	99.6
	Coverage	0.956	0.955	0.955	0.851	0.884
	MCSE (coverage)	0.003	0.003	0.003	0.005	0.005
*n* = 5000	Type I error	0.047	0.048	0.042	0.154	0.055
	MCSE (type I error)	0.003	0.003	0.003	0.005	0.003
	Mean	1	1	1	1	1
	MCSE (mean)	0.001	0.001	0.001	0.001	0.001
	Convergence	100	100	100	100	99.98
	Coverage	0.953	0.952	0.952	0.846	0.878
	MCSE (coverage)	0.003	0.003	0.003	0.005	0.005

*GEE* generalized estimating equations, *MH* Mantel–Haenszel, *RE* random-effects (i.e., random center intercept), *MCSE* Monte Carlo standard errors

**Table 4:** Power, mean estimated odds ratio (OR), convergence rate (%), and coverage of 95% confidence intervals when the number of centers was 100, the ICC was 0.025, the event rate was 10%, the participant distribution across centers was skewed, and the true OR (conditional) was greater than 1

Number of participants (***n***) and true OR	Measurement	Unadjusted	RE	MH	GEE	GEE-small sample correction
*n* = 200 True OR, 3.0	Power	0.805	0.807	NA	0.809	0.791
	MCSE (power)	0.006	0.006	NA	0.006	0.006
	Mean	3.1	3.25	NA	3.1	3.1
	MCSE (mean)	0.006	0.006	NA	0.006	0.006
	Convergence	100	100	0	99.94	99.9
	Coverage	0.958	0.959	NA	0.955	0.958
	MCSE (coverage)	0.006	0.003	NA	0.003	0.003
*n* = 500 True OR, 2.08	Power	0.804	0.807	0.779	0.809	0.794
	MCSE (power)	0.006	0.006	0.038	0.006	0.006
	Mean	2.08	2.11	2.11	2.08	2.08
	MCSE (mean)	0.004	0.004	0.024	0.004	0.004
	Convergence	100	100	2.44	100	100
	Coverage	0.956	0.954	0.959	0.951	0.952
	MCSE (coverage)	0.003	0.003	0.018	0.003	0.003
*n* = 1000 True OR, 1.71	Power	0.803	0.807	0.780	0.806	0.798
	MCSE (power)	0.006	0.006	0.006	0.006	0.006
	Mean	1.7	1.71	1.71	1.7	1.7
	MCSE (mean)	0.003	0.003	0.003	0.003	0.003
	Convergence	100	100	95.94	100	100
	Coverage	0.950	0.949	0.949	0.947	0.949
	MCSE (coverage)	0.003	0.003	0.003	0.003	0.003
*n* = 5000 True OR, 1.29	Power	0.793	0.795	0.783	0.799	0.788
	MCSE (power)	0.006	0.006	0.006	0.006	0.006
	Mean	1.28	1.28	1.28	1.28	1.28
	MCSE (mean)	0.001	0.001	0.001	0.001	0.001
	Convergence	100	100	100	100	100
	Coverage	0.949	0.949	0.949	0.943	0.944
	MCSE (coverage)	0.003	0.003	0.003	0.003	0.003

The average actual number of centers was 87, 99, 100, and 100

*GEE* generalized estimating equations, *MH* Mantel–Haenszel, *RE* random-effects (i.e., random intercept), *MCSE* Monte Carlo standard errors

The Mantel–Haenszel method gave biased estimates, lower than nominal type I error rates, and low power in many scenarios. Further, when the participant distribution across centers was skewed, the number of centers was large, and the number of participants was small, we encountered issues with model convergence.

With a small number of centers, the GEE method produced an inflated type I error rate. This improved with a larger number of centers but was still problematic in some scenarios. Conversely, the GEE-small sample correction approach maintained the type I error rate at the nominal level even when the number of centers was small. However, in these scenarios, the GEE-small sample correction approach had much lower power than RE models. When the number of centers was moderate to large, GEE-small sample correction gave close to nominal type I error rates and good power across all scenarios and performed comparably to RE models.

### Reanalysis of the motivating smoking-cessation trial

The motivation for this work was a five-arm RCT, in which 6006 smokers employed by 54 companies were randomly assigned to one of four smoking cessation interventions or to usual care [3]. The primary outcome was sustained abstinence from smoking through 6 months, achieved by 80 (1.3%) of the 6006 randomized participants (Table 5). The original analysis used a RE model, and the estimated ICC was 0.0045 (95% CI 0.0005 to 0.0117). There are several things to note in the reanalysis shown in Table 5. First, the unadjusted, RE, and GEE models all produced similar results. The GEE-small sample correction model failed to converge. Finally, the Mantel–Haenszel method produced estimates distinctly different from the other models, and also excluded some participants, corresponding with what we observed in the simulation study.

**Table 5:** Reanalysis of the motivating five-arm smoking cessation trial [3]

Study arm^a^	Randomized (*n*)	6-month cessation (*n* (%))	No adjustment	Random intercept	MH	GEE	GEE-small sample correction
Usual care	813	1 (0.1%)	0.12 (0.02 to 0.90)	0.12 (0.02 to 0.90)	0.14 (0.02 to 1.24)	0.14 (0.02 to 0.87)	Model did not converge
Free cessation aids	1587*	8 (0.5%)	0.51 (0.21 to 1.26)	0.51 (0.21 to 1.25)	1.95 (0.81 to 4.7)	0.53 (0.22 to 1.24)	
Free e-cigarettes	1199	12 (1.0%)	Reference	Reference	Reference	Reference	
Rewards plus free cessation aids	1198	24 (2.0%)	2.02 (1.01 to 4.06)	2.02 (1.01 to 4.07)	2.05 (1.02 to 4.14)	1.96 (1.00 to 3.84)	
Redeemable deposit plus free cessation aids	1207*	35 (2.9%)	2.95 (1.52 to 5.71)	2.97 (1.53 to 5.76)	2.96 (1.53 to 5.72)	2.84 (1.50 to 5.37)	
Total	6004 (100%)	80 (1.3%)	6004 (100%)	6004 (100%)	552/2012 (27.44%)^b^ 1025/2786 (36.79%)^b^ 1497/2397 (62.45%)^b^ 1701/2406 (70.70%)^b^	6004 (100%)	

Results are reported as odds ratios and 95% confidence intervals unless otherwise specified. All models are additionally adjusted for study wave (first or second) according to the primary analysis plan

*GEE* generalized estimating equations, *MH* Mantel–Haenszel, *RE* random-effects (i.e., random center intercept)

*A total of 6006 participants were randomized, though for 2 participants (*n* = 1 in each study arm with an asterisk) who did not meet the criteria for the primary outcome, we did not have information on their employer

^a^Details about the interventions examined in this smoking cessation trial are detailed in the primary trial report [3]

^b^Because odds ratios were calculated by each study arm using the e-cigarettes group as the reference group, we have reported the total number for each analysis to illustrate the dropout that occurred when using the Mantel–Haenszel approach

## Discussion

In this paper, we evaluated different methods of estimating an odds ratio in multicenter RCTs with very low event rates, where some centers, or treatment arms within centers, experienced no outcome events. We focused on five popular methods of analysis: (i) no center adjustment, (ii) RE, (iii) Mantel–Haenszel model, (iv) GEE, and (v) GEE-small sample correction. From our simulation results, we found that RE models performed well overall, and we recommend their use in most settings. GEE-small sample correction models worked well when there was a moderate to large number of centers, while GEE still had a slightly inflated type I error rate, especially when the number of centers was 50 or the total participant number was large (i.e., 5000). When the total number of participants in a trial is small but the number of centers is high, RE models may give biased estimates. Thus, GEE or GEE-small sample correction models could be considered in such situations. Further, based on our difficulties with model convergence, we suggest that researchers include an alternative method in their statistical analysis plans in case the primary method fails to converge. Table 6 provides selected recommendations according to the number of centers.

**Table 6:** Selected recommendations for writing a statistical analysis plan for a multicenter trial where sparse or no events may occur within a center or stratification variable

Number of centers	RE	MH	GEE	GEE-small sample correction
Small number of centers (e.g., 5)	Recommended	X Low type I error and power	X Inflated type I error	X Nominal type I error, but low power Convergence issues
Moderate or large number of centers (e.g., 50 to 100)	Recommended (slightly biased odds ratio with a small total sample size (e.g., 200))	X Low type I error and power Convergence issues when the sample size is small (e.g., 200)	△ Slightly inflated type I error in some scenarios	Recommended

*GEE* generalized estimating equations, *MH* Mantel–Haenszel, *RE* random-effects (i.e., random center intercept)

X: We do not recommend this method

△: Consider other methods or use with caution

In addition to the simulation study, we reanalyzed the RCT that motivated this paper. The sparsity of the 80 events across the five study arms and 54 companies by which randomization was stratified produced a setting where the choice of analysis is very important in order to avoid unnecessarily dropping many participants from the analysis. We also encountered challenges with convergence for some of the treatment contrasts when using the Mantel–Haenszel model and the GEE-small sample correction. The results for the center unadjusted, GEE, and RE models were all similar. These results are likely due to the very low ICC (0.005). Of note, the use of a fixed-effects analysis would result in 1116 (18.6%) randomized participants being excluded from the analysis.

Our study has limitations. First, we studied commonly used methods, as we believe that recommendations stemming from these comparisons will be most informative to a broad clinical and social science audience. However, there are other potentially useful methods that could be used, particularly Bayesian methods and Firth’s correction. Readers interested in Bayesian options for the analysis of multicenter trials with binary outcomes are directed to the work by Pedroza and Truong [6]. In their simulation study of model performance in studies with less than or equal to 30 centers and 50 or fewer participants per center, they compared GEE log-binomial and Poisson models, generalized linear mixed models (GLMMs) assuming binomial and Poisson distributions, and a Bayesian binomial GLMM with informative neutral priors. They observed that all of the examined frequentist methods exhibited little bias and a similar root mean squared error (RMSE). They noted convergence issues with some models, including the Bayesian binomial GLMM informative neutral priors but found that this method had the smallest RMSE and good coverage across all scenarios. Heinze and Schemper [21] showed that Firth’s modification of the score function was a tenable option in analyses of two different clinical datasets. Agresti and Hartzel [4] also reviewed the pros and cons of other strategies that researchers might consider, such as combining low or no event centers. Second, we did not examine questions related to treatment-by-center interaction. However, this topic has also been reviewed by Agresti and Hartzel [4]. Third, we simulated data from a random intercept model. Therefore, it is likely that the results favor analysis using this model.

To conclude, we examined a range of hypothetical trial settings where randomization was stratified by center, but in certain centers, there were no outcome events overall or in one study arm of the stratum. This simulation study was motivated by our experience with a completed trial. Accordingly, we provide selected recommendations for the analysis of multicenter RCTs where randomization has been stratified by center in Table 6. Our findings generally align with the suggestions and findings of prior work regarding the analysis of multicenter RCTs where the primary outcome is binary [4–7]. Specifically, we found that random intercept models generally performed best across most scenarios. GEE-small sample correction performed well when the number of centers was large. We do not recommend the use of Mantel–Haenszel, GEE, or models that do not account for center. When the planned event rate is low, we suggest that the statistical analysis plan specify an alternative method in the case of non-convergence of the primary method. The issues demonstrated in this article should highlight the care that is needed to ensure a statistical analysis plan that is resilient to unanticipated data distributions or unexpected non-convergence of statistical models.

## Supplementary information

**Additional file 1: Table S1**. Calculated true odds ratio when OR > 1. **Figure S1**. Type I error rates for scenarios when then the number of centers is 5, 50, and 100, the total patients are 200, 500, 1000, and 5000. **Figure S2**. Power for scenarios when the number of centers is 5, 50, and 100, the total patients are 200, 500, 1000, and 5000. **Figure S3**. Estimated mean treatment OR for scenarios when the true odds ratio is 1, the number of centers is 5, 50, and 100, the total patients are 200, 500, 1000, and 5000. **Figure S4**. Coverage of 95% CIs for scenarios when the true odds ratio is 1, the number of centers is 5, 50, and 100, the total patients are 200, 500, 1000, and 5000. **Figure S5**. Coverage of 95% CIs for scenarios when the true odds ratio is greater than 1, the number of centers is 5, 50, and 100, the total patients are 200, 500, 1000, and 5000. **Figure S6**. Type I error rates for scenarios when then the number of centers is 5, 50, and 100, the total patients are 200, 500, 1000, and 5000. **Figure S7**. Power for scenarios when the number of centers is 5, 50, and 100, the total patients are 200, 500, 1000, and 5000. **Figure S8**. Estimated mean treatment OR for scenarios when the true odds ratio is 1, the number of centers is 5, 50, and 100, the total patients are 200, 500, 1000, and 5000. **Figure S9**. Coverage of 95% CIs for scenarios when the true odds ratio is 1, the number of centers is 5, 50, and 100, the total patients are 200, 500, 1000, and 5000. **Figure S10**. Coverage of 95% CIs for scenarios when the true odds ratio is greater than 1, the number of centers is 5, 50, and 100, the total patients are 200, 500, 1000, and 5000. **Figure S11**. Type I error rates for scenarios when then the number of centers is 5, 50, and 100, the total patients are 200, 500, 1000, and 5000. **Figure S12**. Power for scenarios when the number of centers is 5, 50, and 100, the total patients are 200, 500, 1000, and 5000. **Figure S13**. Estimated mean treatment OR for scenarios when the true odds ratio is 1, the number of centers is 5, 50, and 100, the total patients are 200, 500, 1000, and 5000. **Figure S14**. Coverage of 95% CIs for scenarios when the true odds ratio is 1, the number of centers is 5, 50, and 100, the total patients are 200, 500, 1000, and 5000. **Figure S15**. Coverage of 95% CIs for scenarios when the true odds ratio is greater than 1, the number of centers is 5, 50, and 100, the total patients are 200, 500, 1000, and 5000.

## Data Availability

The datasets used and/or analyzed during the current study are available from the corresponding author upon reasonable request.
