# Supplementary material for: Analysis of multicenter clinical trials with very low event rates
Source: Trials. 2020 Nov 9;21:917. doi: 10.1186/s13063-020-04801-5 (PMC7654615; doi:10.1186/s13063-020-04801-5)
Supplement: Supplementary file 1 — Additional file 1: Table S1. Calculated true odds ratio when OR > 1. Figure S1. Type I error rates for scenarios when then the number of centers is 5, 50, and 100, the total patients are 200, 500, 1000, and 5000. Figure S2. Power for scenarios when the number of centers is 5, 50, and 100, the total patients are 200, 500, 1000, and 5000. Figure S3. Estimated mean treatment OR for scenarios when the true odds ratio is 1, the number of centers is 5, 50, and 100, the total patients are 200, 500, 1000, and 5000. Figure S4. Coverage of 95% CIs for scenarios when the true odds ratio is 1, the number of centers is 5, 50, and 100, the total patients are 200, 500, 1000, and 5000. Figure S5. Coverage of 95% CIs for scenarios when the true odds ratio is greater than 1, the number of centers is 5, 50, and 100, the total patients are 200, 500, 1000, and 5000. Figure S6. Type I error rates for scenarios when then the number of centers is 5, 50, and 100, the total patients are 200, 500, 1000, and 5000. Figure S7. Power for scenarios when the number of centers is 5, 50, and 100, the total patients are 200, 500, 1000, and 5000. Figure S8. Estimated mean treatment OR for scenarios when the true odds ratio is 1, the number of centers is 5, 50, and 100, the total patients are 200, 500, 1000, and 5000. Figure S9. Coverage of 95% CIs for scenarios when the true odds ratio is 1, the number of centers is 5, 50, and 100, the total patients are 200, 500, 1000, and 5000. Figure S10. Coverage of 95% CIs for scenarios when the true odds ratio is greater than 1, the number of centers is 5, 50, and 100, the total patients are 200, 500, 1000, and 5000. Figure S11. Type I error rates for scenarios when then the number of centers is 5, 50, and 100, the total patients are 200, 500, 1000, and 5000. Figure S12. Power for scenarios when the number of centers is 5, 50, and 100, the total patients are 200, 500, 1000, and 5000. Figure S13. Estimated mean treatment OR for scenarios when the true odds ratio i [file 13063_2020_4801_MOESM1_ESM.docx]

**Online supplement**

**for**

**“Analysis of multicenter clinical trials with very low event rates”**

Authors:

Jiyu Kim, Andrea B. Troxel, Scott D. Halpern, Kevin G. Volpp,

Brennan C. Kahan, Tim P. Morris, Michael O. Harhay

Table of Contents

[Extended Results 3](#_Toc26947226)

[5 centers 3](#_Toc26947227)

[50 centers 4](#_Toc26947228)

[100 centers 5](#_Toc26947229)

[Additional figures and table 6](#_Toc26947230)

[Table S1. Calculated true odds ratio when OR>1 6](#_Toc26947231)

[Results: The patient distribution is even and ICC is 0.025 7](#_Toc26947232)

[**Figure S1**. Type I error rates for scenarios when then the number of centers is 5, 50, and 100, the total patients are 200, 500, 1000, and 5000. 7](#_Toc26947233)

[**Figure S2**. Power for scenarios when the number of centers is 5, 50, and 100, the total patients are 200, 500, 1000, and 5000. 8](#_Toc26947234)

[**Figure S3**. Estimated mean treatment OR for scenarios when the true odds ratio is 1, the number of centers is 5, 50, and 100, the total patients are 200, 500, 1000, and 5000. 9](#_Toc26947235)

[**Figure S4**. Coverage of 95% CIs for scenarios when the true odds ratio is 1, the number of centers is 5, 50, and 100, the total patients are 200, 500, 1000, and 5000. 10](#_Toc26947236)

[**Figure S5**. Coverage of 95% CIs for scenarios when the true odds ratio is greater than 1, the number of centers is 5, 50, and 100, the total patients are 200, 500, 1000, and 5000. 11](#_Toc26947237)

[Results: The patient distribution is skewed and ICC is 0.075 12](#_Toc26947238)

[**Figure S6**. Type I error rates for scenarios when then the number of centers is 5, 50, and 100, the total patients are 200, 500, 1000, and 5000. 12](#_Toc26947239)

[**Figure S7**. Power for scenarios when the number of centers is 5, 50, and 100, the total patients are 200, 500, 1000, and 5000. 13](#_Toc26947240)

[**Figure S8**. Estimated mean treatment OR for scenarios when the true odds ratio is 1, the number of centers is 5, 50, and 100, the total patients are 200, 500, 1000, and 5000. 14](#_Toc26947241)

[**Figure S9**. Coverage of 95% CIs for scenarios when the true odds ratio is 1, the number of centers is 5, 50, and 100, the total patients are 200, 500, 1000, and 5000. 15](#_Toc26947242)

[**Figure S10**. Coverage of 95% CIs for scenarios when the true odds ratio is greater than 1, the number of centers is 5, 50, and 100, the total patients are 200, 500, 1000, and 5000. 16](#_Toc26947243)

[Results: The patient distribution is even and ICC is 0.075 17](#_Toc26947244)

[**Figure S11**. Type I error rates for scenarios when then the number of centers is 5, 50, and 100, the total patients are 200, 500, 1000, and 5000. 17](#_Toc26947245)

[**Figure S12**. Power for scenarios when the number of centers is 5, 50, and 100, the total patients are 200, 500, 1000, and 5000. 18](#_Toc26947246)

[**Figure S13**. Estimated mean treatment OR for scenarios when the true odds ratio is 1, the number of centers is 5, 50, and 100, the total patients are 200, 500, 1000, and 5000. 19](#_Toc26947247)

[**Figure S14**. Coverage of 95% CIs for scenarios when the true odds ratio is 1, the number of centers is 5, 50, and 100, the total patients are 200, 500, 1000, and 5000. 20](#_Toc26947248)

[**Figure S15**. Coverage of 95% CIs for scenarios when the true odds ratio is greater than 1, the number of centers is 5, 50, and 100, the total patients are 200, 500, 1000, and 5000. 21](#_Toc26947249)

[R code 22](#_Toc26947250)

# **Extended Results**

The primary text provides an overall summary of key findings for the simulation study. To better aid researchers with a fixed number of centers, we also provide a summary of the simulation study by the total number of centers below.

## 5 centers

All methods gave unbiased estimates of the treatment effect, except for a few scenarios when the total sample size was very small (200 or 500); in this case, all methods were slightly biased. Across all scenarios, GEE gave inflated type I error rates (8-17%). Accordingly, it gave higher power (range 82% to 87%), and this might be because the SEs are underestimated, indicating that GEE would inappropriate to use when the number of centers is small. GEE-small sample correction maintained the type I error rates at the nominal level even when the number of centers was as small as 5, but the power was considerably reduced (range 33% to 60%). Both GEE and GEE-small sample correction showed very low coverage rates in many cases (below 90%).

MH had low type I error rates (range 0.1% to 4%) and power (range 74% to 80%) across all scenarios especially when the total sample size was between 200 and 1000. Although RE and GEE-small sample correction also had low type I error rates when the event rate was extremely small (2%) or the total sample size was smaller than 500, both methods showed nominal value of type I error rate otherwise.

RE generally gave nominal type I error rate and power as total patient is increasing. RE and GEE generally had high convergence rates (above 98%). GEE-small sample correction had the lowest convergence rate overall. (e.g., 89% with 2% event rate and 200 total sample).

The model without adjustment (i.e. logistic regression without adjustment) gave comparable type I error rate and power when the ICC was small (0.025). However, it gave lower type I error rate and power than RE, GEE or GEE-small sample correction in many scenarios with larger ICC (i.e., 0,075).

## 50 centers

Overall, all methods gave unbiased OR, but they showed small bias with some scenarios when the total number of patients was 200.

All methods gave type I error rates that were too low when the total number of patients was 200. GEE and GEE-small sample correction gave nominal value of error rate as the total number increased, but GEE still gave slightly inflated type I error rate (up to 0.066) in some scenarios, while GEE-small sample correction gave reasonable values.

With OR greater than 1, all three methods (RE, GEE and GEE-small sample correction) had comparable power, but RE and GEE had higher power (0.78-0.83) than GEE-small sample correction (0.73 to 0.81) when ICC was 0.025.

Convergence rates for RE, GEE, and GEE-small sample correction were high in most scenarios (above 98%) except when the event rate was 2% (92% at the lowest)

MH gave very low type I error rates in most scenarios, and power also was low when the total sample size was less than 1000. In addition, especially when the patient distribution was skewed across the centers, MH failed to converge in many data sets because of sparseness, leading to a biased estimate of the odds ratio of treatment effect in scenarios with only 200 patients in total.

The model without adjustment (i.e. logistic regression without adjustment) gave quite low type I error rate, which was prominent when ICC is higher (i.e. 0.075). It also gave lower power than RE or GEE, overall.

## 100 centers

RE, GEE and GEE-small sample correction had close to nominal type I error rates in general, except in some scenarios with 2% event rate and 200 or 500 total patients. Those three methods also gave unbiased odds ratio of treatment overall, but RE gave biased estimate when sample size was small (200) with larger Monte Carlo standard error.

Although RE, GEE, and GEE-small sample correction had comparable power across the scenarios close to 80% when the event rate was 10%, they had slightly higher power when the event rate dropped to 5% or 2%. This was more pronounced with ICC of 0.075. On the other hand, all methods lost power with the ICC of 0.025, event rate of 2%, and 200 total patients.

Convergence rates for the model without adjustment, RE, GEE, and GEE-small sample correction were very high across the scenarios (99% or higher). MH also generally had high convergence rates (95.9% or higher) when the total patients were 1000 or higher, but failed to converge often with only 200 or 500 total patients. As with 50 centers, MH gave low type I error all across the scenarios, and lost power when the total number of patients was less than 1000. Especially with total patients of 200, the power dropped to 39-53%, which is also leading to biased estimates.

Similar to 50 centers, the model without adjustment (i.e. logistic regression without adjustment) gave low type I error even though it gave unbiased estimate Also, it gave lower power than RE and GEE model in most of the scenarios.

# **Additional figures and table**

## Table S1. Calculated true odds ratio when OR>1

| Total patient | Event rate | True OR |
| --- | --- | --- |
| 200 | 2% | 6.730 |
| 500 | 2% | 3.831 |
| 1000 | 2% | 2.755 |
| 5000 | 2% | 1.656 |
| 200 | 5% | 4.011 |
| 500 | 5% | 2.584 |
| 1000 | 5% | 2.020 |
| 5000 | 5% | 1.402 |
| 200 | 10% | 3.000 |
| 500 | 10% | 2.077 |
| 1000 | 10% | 1.708 |
| 5000 | 10% | 1.286 |

##

## Results: The patient distribution is even and ICC is 0.025

### **Figure S1**. Type I error rates for scenarios when then the number of centers is 5, 50, and 100, the total patients are 200, 500, 1000, and 5000.


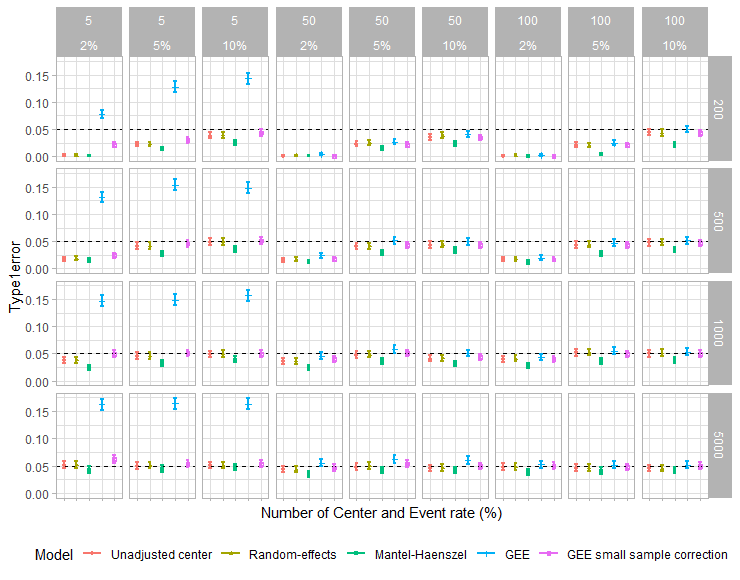


### **Figure S2**. Power for scenarios when the number of centers is 5, 50, and 100, the total patients are 200, 500, 1000, and 5000.

*
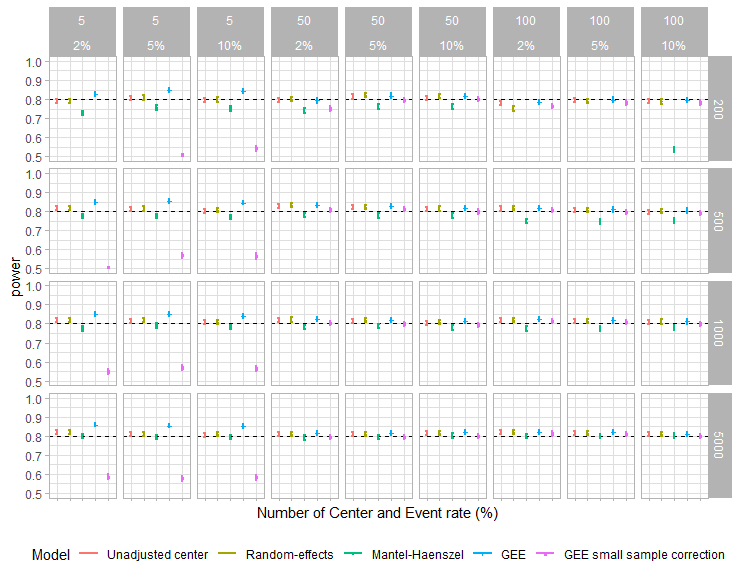
*

*Power for MH when center=100, total patient number=200 were smaller than 0.5 (0.39 and 0.49 with 2%, 5% event rate, respectively.)

### **Figure S3**. Estimated mean treatment OR for scenarios when the true odds ratio is 1, the number of centers is 5, 50, and 100, the total patients are 200, 500, 1000, and 5000.

**
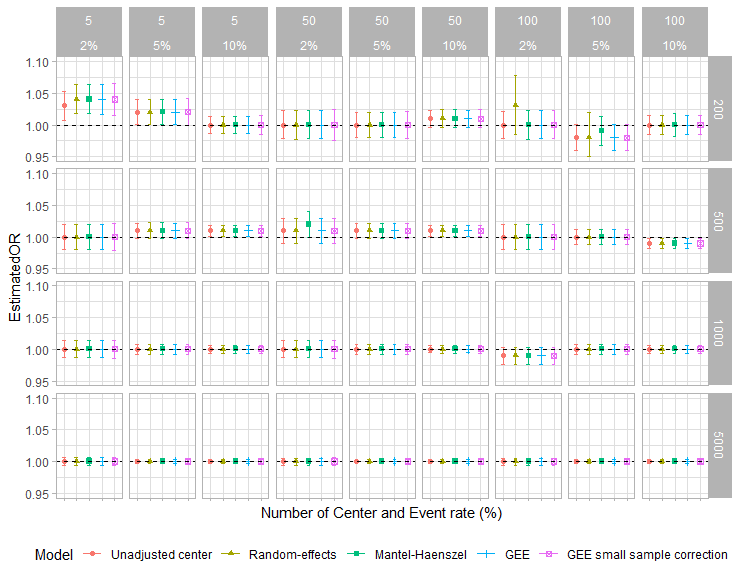
**

### **Figure S4**. Coverage of 95% CIs for scenarios when the true odds ratio is 1, the number of centers is 5, 50, and 100, the total patients are 200, 500, 1000, and 5000.

*
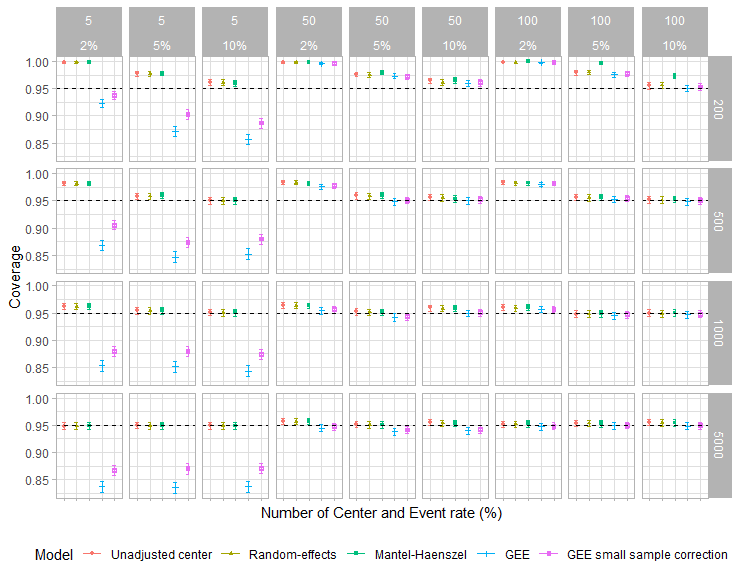
*

### **Figure S5**. Coverage of 95% CIs for scenarios when the true odds ratio is greater than 1, the number of centers is 5, 50, and 100, the total patients are 200, 500, 1000, and 5000.

*
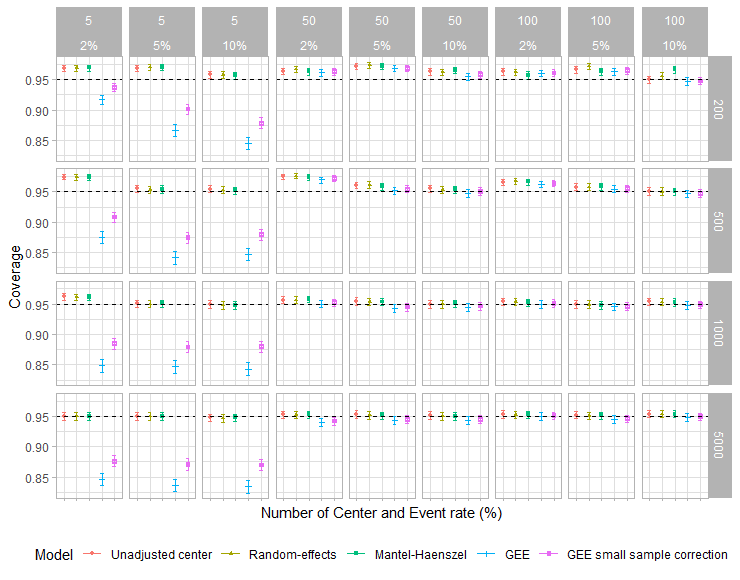
*

## Results: The patient distribution is skewed and ICC is 0.075

### **Figure S6**. Type I error rates for scenarios when then the number of centers is 5, 50, and 100, the total patients are 200, 500, 1000, and 5000.


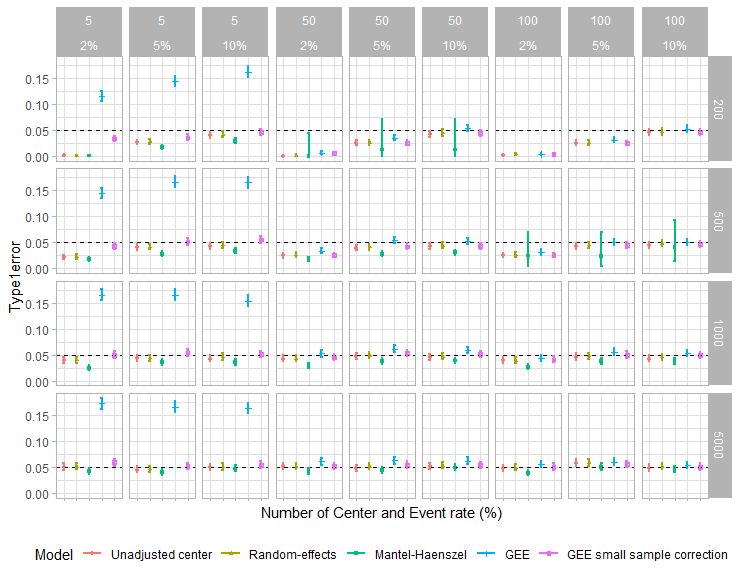


*The value for MH were not available when center=100, total patient number=200.

### **Figure S7**. Power for scenarios when the number of centers is 5, 50, and 100, the total patients are 200, 500, 1000, and 5000.

*
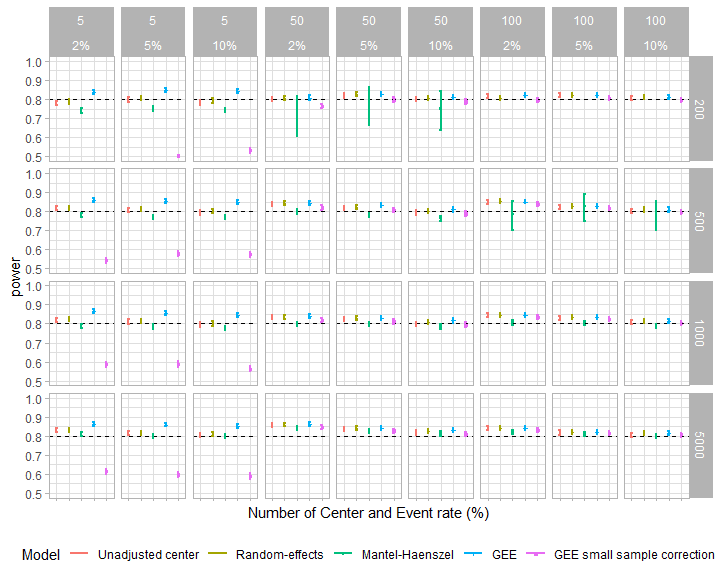
*

*The value for MH were not available when center=100, total patient number=200.

### **Figure S8**. Estimated mean treatment OR for scenarios when the true odds ratio is 1, the number of centers is 5, 50, and 100, the total patients are 200, 500, 1000, and 5000.

**
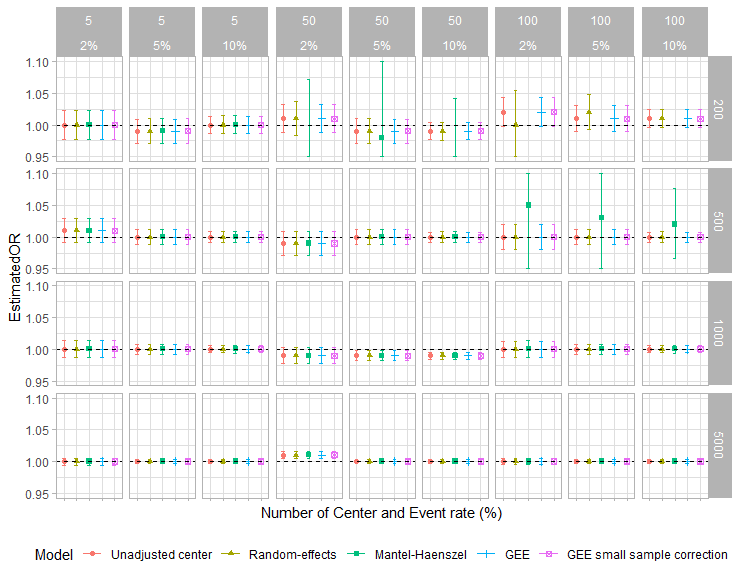
**

*The value for MH were not available when center=100, total patient number=200.

### **Figure S9**. Coverage of 95% CIs for scenarios when the true odds ratio is 1, the number of centers is 5, 50, and 100, the total patients are 200, 500, 1000, and 5000.

*
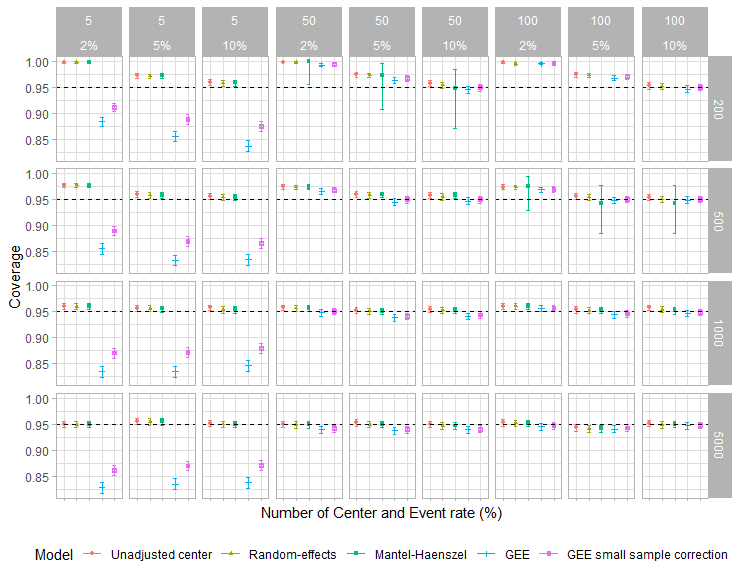
*

*The value for MH were not available when center=100, total patient number=200.

### **Figure S10**. Coverage of 95% CIs for scenarios when the true odds ratio is greater than 1, the number of centers is 5, 50, and 100, the total patients are 200, 500, 1000, and 5000.

**
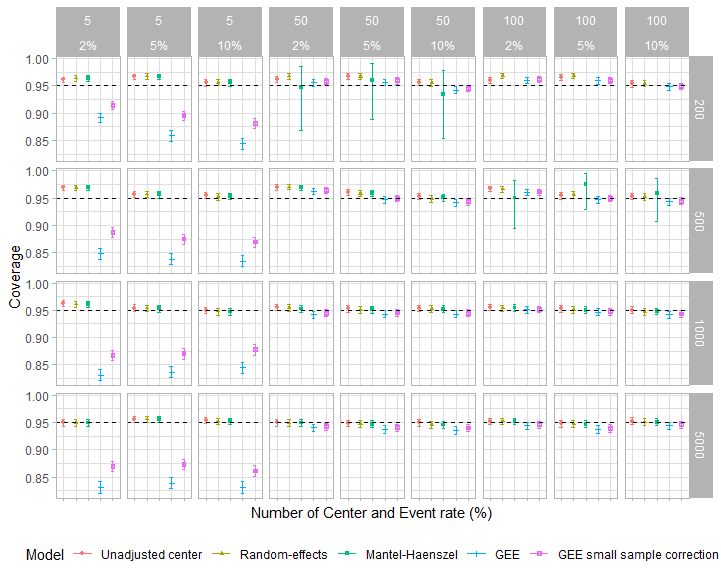
**

*The value for MH were not available when center=100, total patient number=200.

## Results: The patient distribution is even and ICC is 0.075

### **Figure S11**. Type I error rates for scenarios when then the number of centers is 5, 50, and 100, the total patients are 200, 500, 1000, and 5000.


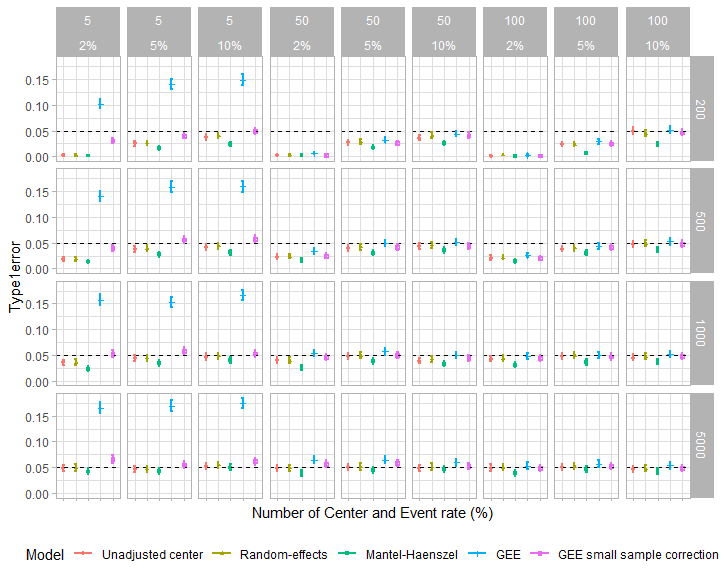


### **Figure S12**. Power for scenarios when the number of centers is 5, 50, and 100, the total patients are 200, 500, 1000, and 5000.

*
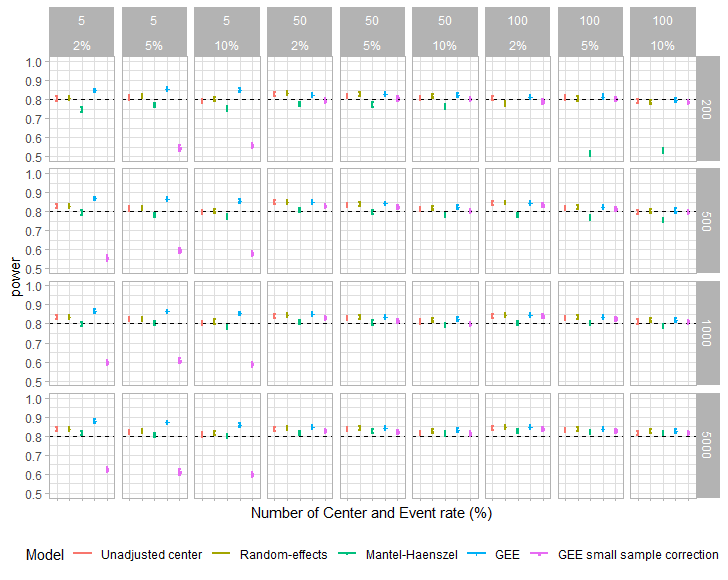
*

*The power for MH when center=100, total patients=200 were 0.43.

### **Figure S13**. Estimated mean treatment OR for scenarios when the true odds ratio is 1, the number of centers is 5, 50, and 100, the total patients are 200, 500, 1000, and 5000.


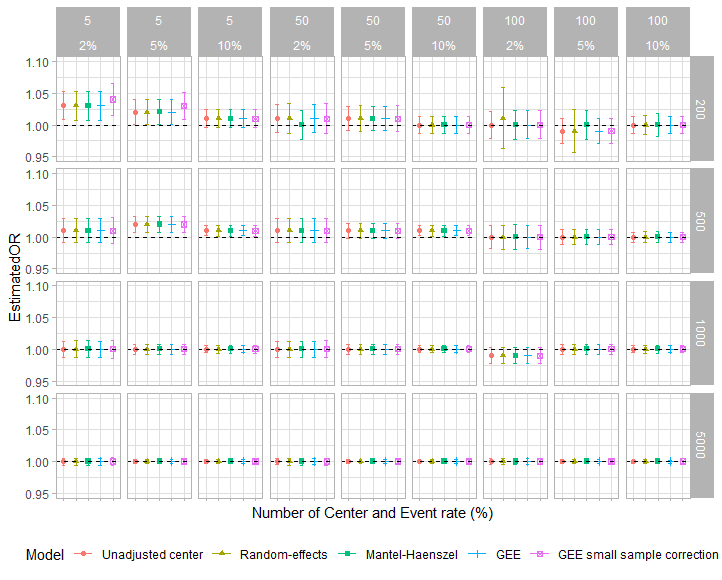


### **Figure S14**. Coverage of 95% CIs for scenarios when the true odds ratio is 1, the number of centers is 5, 50, and 100, the total patients are 200, 500, 1000, and 5000.

*
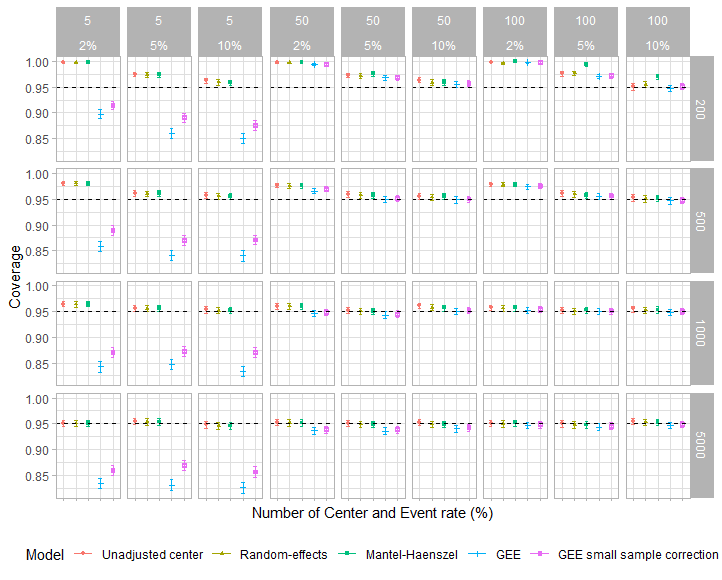
*

### **Figure S15**. Coverage of 95% CIs for scenarios when the true odds ratio is greater than 1, the number of centers is 5, 50, and 100, the total patients are 200, 500, 1000, and 5000.

*
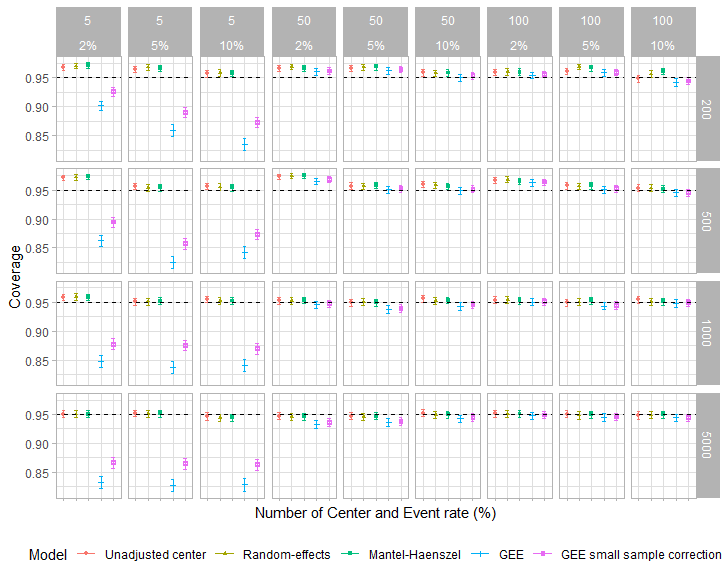
*

# **R code**

#Data generating

for(j in 1:length(strat)){

r<-blockrand(n=nPerStrat, levels=c('T', 'C'),

id.prefix=stratn[j], block.sizes=bs)

r<-r[1:(TNpatient/center),]

d<-rbind(d, r)

}

alpha<-log(pc/(1-pc))

ruj<-rnorm(center, 0, centvar)

#centvar; calculated from ICC value (ICC=0.025:0.2904, ICC=0.075: 0.5165)

uj<-rep(ruj, nPerStrat)

ej<-rlogis(TNpatient, 0, 1)

cent<-rep(1:center, nPerStrat)

cu<-data.frame(alpha,uj,ej,cent,beta)

cu<-cu[order(cent),]

d<-cbind(d,cu)

d$trt<-ifelse(d$treatment=='T',1,0)

d$ystar <- alpha+d$beta*d$trt+d$uj+d$ej

d$yij<-ifelse(d$ystar>0,1,0)

Res[[i]]<-d #ith dataset

#Unadjusted center#

F.test<-glm(yij~trt, data=data, family=binomial(link='logit'))

#Random effect model#

R.test<-glmer(yij~trt+(1|cent), data=data, family=binomial(link='logit'), nAGQ = 9)

#MH-Test #

MHtable<-as.data.frame(table(data))

Table = xtabs(Freq ~ trt+ yij+ cent, data=MHtable)

MH.test<-mantelhaen.test(Table)

#GEE model#

G.test<-geeglm(yij~trt, id=cent, family=binomial(link='logit'), data=data, corstr = 'exchangeable')

#GEE-small sample correction model#

GC.test<-gee(yij~trt, id=cent, data=data, family=binomial, corstr = "exchangeable")

u<-geeUOmega(GC.test)

saws(u,method="d5")
